# Two Individual Super-Bound State Modes within Band Gap with Ultra-High *Q* Factor for Potential Sensing Applications in the Terahertz Wave Band

**DOI:** 10.3390/s23156737

**Published:** 2023-07-27

**Authors:** Yinbing An, Tao Fu, Chunyu Guo, Jihong Pei, Zhengbiao Ouyang

**Affiliations:** 1 College of Physics and Optoelectronic Engineering, Shenzhen University, Shenzhen 518060, Chinacyguo@szu.edu.cn (C.G.); 2 THz Technical Research Center, Shenzhen Key Laboratory of Micro-Nano Photonic Information Technology, Key Laboratory of Optoelectronic Devices and Systems of Ministry of Education and Guangdong Province, Shenzhen University, Shenzhen 518060, China; 3 Guangxi Key Laboratory of Precision Navigation Technology and Application, Guilin University of Electronic Technology, Guilin 541004, China; 4 College of Electronics and Information Engineering, Shenzhen University, Shenzhen 518060, China; jhpei@szu.edu.cn

**Keywords:** refractive index sensor, multi-wavelength sensor, bound state in continuum, super bound state, photonic band gap

## Abstract

Bound states in the continuum (BICs) garnered significant research interest in the field of sensors due to their exceptionally high-quality factors. However, the wide-band continuum in BICs are noise to the bound states, and it is difficult to control and filter. Therefore, we constructed a top-bottom symmetric cavity containing three high permittivity rectangular columns. The cavity supports a symmetry-protected (SP) superbound state (SBS) mode and an accidental (AC) SBS mode within the bandgap. With a period size of 5 × 15, the bandgap effectively filters out the continuum, allowing only the bound states to exist. This configuration enabled us to achieve a high signal-to-noise ratio and a wide free-spectral-range. The AC SBS and the SP SBS can be converted into quasi-SBS by adjusting different parameters. Consequently, the cavity can function as a single-band sensor or a dual-band sensor. The achieved bulk sensitivity was 38 µm/RIU in terahertz wave band, and a record-high FOM reached 2.8 × 10^8^ RIU^−1^. The effect of fabrication error on the performance for sensor application was also discussed, showing that the application was feasible. Moreover, for experimental realization, a 3D schematic was presented. These achievements pave the way for compact, high-sensitivity biosensing, multi-wavelength sensing, and other promising applications.

## 1. Introduction

Bound states in continuums (BICs) attracted significant attention for open systems without coupling channels from outside radiation, as they possess infinite *Q*-factors and have become a hot topic of research in interfering systems [1,2], extending from quantum mechanics [3] to photonics [1,2,3,4]. BICs can be divided into several groups based on their intrinsic topological nature, with two of the most conventional kinds being symmetry-protected (SP) BICs, and accidental (AC) BICs [5]. An symmetry-protected (SP) BIC exists in a system that exhibits mirror or rotational symmetry, and this BIC mode belongs to different symmetry classes that completely decouple with the system as long as the symmetry is preserved [1], while an accidental (AC) BIC is a single resonance that evolves into a BIC when enough parameters are tuned. The single resonance itself can be thought of as arising from two (or more) sets of waves, and the radiation of the constituting waves can be tuned to cancel each other [1]. The BIC represents a perfectly localized state with no leakage energy, even when it coexists with a continuous spectrum of radiating waves, which effectively manipulates the light-matter interaction and generates an ultrahigh *Q*-factor resonance [1,2]. Thus, BICs found prospects in various material systems of photonics crystals [6,7,8], topological insulators [9,10], and metamaterials [11,12,13].

The optical BIC is invisible with zero linewidth and an infinite *Q*-factor in its optical spectra [14]. When it collapses to the quasi-BIC (QBIC), it can be experimentally observed with a highly enhanced *Q* factor [14,15,16,17] and other interesting phenomena [1,2]. These unique features distinguish BICs from the traditional optical modes and significantly improve the performance of optical devices, especially for sensors, whose sensitivity is directly affected by the *Q* factor. In recent years, BICs were well explored in the literature for sensors in different structures, such as photonic crystals [7,18,19], metasurfaces [20,21,22,23,24,25,26,27,28,29,30,31,32,33,34,35], and gratings [36,37,38,39]. These BIC sensors have different uses, for instance, refractive index sensing [7,25,28,30,36,39,40], biosensing [20,34,41,42], and gas detection [37], ranging from visible [37] to infrared [23,33,38,39] to THz [21,22,29,31,35,43] regions.

However, BICs are locked in the passband of the continua in an open system, and these continua can be regarded as noise, limited the free-spectral-range (FSR, namely, there exists only one resonance within a broad optical span) of the sensors, and the FSR directly limits the operation span. Nevertheless, limited by the finite periodic system [44], or fabrication errors [45], the radiative loss is difficult to suppress, and only QBICs with finite radiative lifetime can be actually observed, exhibiting Fano resonances in the scattering spectrum [14,15,16,17,46]. The *Q*-factors of most QBICs are affected by the dimension of the open systems [8,47]; the larger the period, the higher the *Q*-factor. Obtaining high *Q*-values with small structural sizes is, therefore, challenging. 

Therefore, the design of ultra-high *Q* SBS modes in the bandgap is of interest for sensing. These SBSs exhibit quality factors that approach infinity and are free from the background noise of the continuum. The characteristics of SBSs and BICs are the same; however, an SBS exists within a bandgap, whereas a BIC is in a continuum of waves. The band gap of the SBS structure effectively filters out the background continuum. This is an important difference between them and produces a considerable advantage for the SBSs over BICs that the SBSs are with much wider FSR and high signal-to-noise ratio than BICs.

In the previous work [48], we employed a two-dimensional photonic crystal (PhC) cavity with one rectangular defect column to generate SP SBSs. In this study, we changed the cavity with three defect columns, the new cavity can generate both accidental (AC) and SP SBSs, and the *Q* factor improved almost 100 times with the same period size. The cavity had top-bottom symmetry and supported an inverse-phase field pattern (SP SBS mode) or dipole field (AC SBS mode). 

The quality factors of these SBSs are theoretically as infinite as those of BICs. Moreover, there is no stray interference frequency component in the wide frequency range next to the SBS modes, which is conducive to obtaining an ultra-wide FSR; thus, they have a larger operation span. However, ideal SBSs are not visible, which again limits their application. The symmetry can be slightly broken for SP SBS and the parameters of the AC SBS can be tuned away from the SBS point to obtain quasi-SBSs (QSBSs) that are visible at the output port and have limited albeit very high-quality factors; thus, they can be used for sensor applications. 

In this paper, we studied the transmission spectrum of an SP SBS and an AC SBS within bandgap. The calculations indicate that, for one thing, the bandgap can filter out the background light, allowing the QSBS to have Lorentz line types in addition to being limited to Fano resonance, thus expanding its range of applications. Additionally, the two SBSs in bandgap can be modulated individually, we can obtain a single AC QSBS or SP QSBS in the transmission spectral, and we can also obtain both in the transmission spectral by tuning the geometric parameter. A top-bottom symmetric super-cavity with an SP SBS mode and an AC SBS mode in the bandgap was designed. The two SBSs resulted in an extremely high *Q*-factor that was more than 10^10^ with a period size of 5 × 15; thus, we can obtain an ultra-high FOM that is more than 10^8^. These research findings have a positive impact on the miniaturization of high-quality factor sensors.

The remainder of this paper is organized as follows. In Section 2, the property of the AC SBSs and SP SBSs are presented. In Section 3, the sensing property of the AC SBSs and SP SBSs are shown. Finally, Section 4 presents the conclusions of this study.

## 2. Materials and Methods

**Figure 1:**
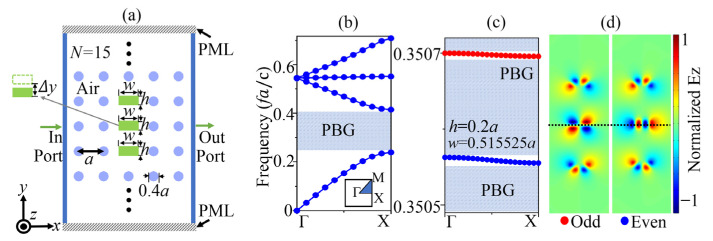
(**a**) Schematic of the PhC cavity structure. (**b**) TE band map of the simplified 2D perfect PhC along the Γ-X direction. (**c**) TE Band maps and (**d**) electrical field patterns in z-direction of the SBS modes when h = 0.2a, w = 0.515525a in the bandgap along the Γ-X direction.

**Figure 2:**
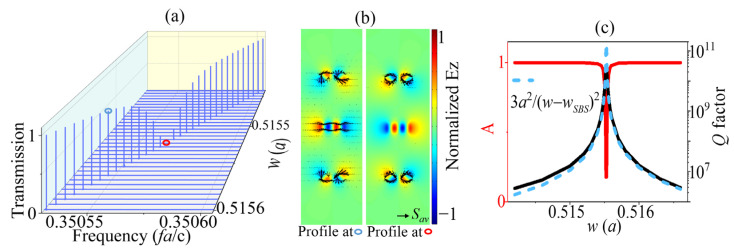
The evolution of (**a**) transmission spectral profile and (**c**) *Q* factors (black line) and amplitude (*A*, red line) of the AC SBS mode with different w values when h = 0.2a. (**b**) Electrical field patterns in z-direction of the defect cavity in the SBS point and QSBS point.

Figure 1a shows the schematics and parameter symbols of the proposed structure. Circular cylinders of 5 × 15 were arranged in air to form a two-dimensional PhC and the central three circular columns were replaced with rectangular columns (defect columns). The height of the columns in the z direction was infinite. A TE plane wave with an electric field in the *z*-direction propagated from the port “in” to the port “out” along the x-direction with wave vector $k_{x}$. The lattice constant of the square lattice PhC was a and dielectric silicon columns had permittivity ε = 12.25. The three rectangular rutile ceramics pillars at the center, with permittivity ε = 100, are marked in green. Additionally, the width (w) and height (h) of the rectangular defect pillars were the same, and w is the most important parameter for achieving the AC SBS. The center defect column can be moved in *y*-direction, the amount of movement is Δy, which is the most significant parameter to tune SP SBS. Two ports marked as the blue solid line indicate the input and output ports of the wave for the cavity. In Figure 1b, the band map of the simplified 2D perfect PhC was obtained using the finite element method. The ideal silicon PhC exhibited a large photonic bandgap in the range of 0.2393–0.4149 in units of fa/c (c is the light speed in vacuum). The AC SBS (even mode in blue line) and SP SBS (odd mode in red line) modes in the photonic bandgap are shown in Figure 2c,d with h = 0.2a, w = 0.515525a. The band map of the cavity was calculated by treating the entire structure as a supercell in the finite element method.

Figure 2 shows the transmission and *Q*-factor properties of the AC SBS mode. In Figure 2a, the evolution of the transmission illustrates that the amplitude of the peak nearly disappeared at ($w_{SBS}$ = 0.515525a) the SBS point. The other peaks of those transmissions decreased from both sides of the SBS point with w closing to 0.515525a. The field profiles and Poynting vector for the SBS point (the red circle in Figure 2a) and a typical w (the blue circle in Figure 2a) were chosen to show the difference between them. The two field patterns had minor differences, while the Poynting vectors showed a significant variation in the center. For the profile of the blue circle point in the left side panel of Figure 2b, the Poynting vectors indicate that the energy radiated to the out space of the cavity. However, for the other panel of Figure 2b, there was no radiation energy from the center field of the BIC point, and the up and down parts had reverse relation, giving no radiating channel to the free space. In momentum space, *Q* is shown to decay quadratically ($Q\propto k^{-2}$) with respect to the distance k from a single isolated BIC [49]. Here, there is a similar phenomenon: when w tends to $w_{SBS}$, *Q* increases quadratically, or $Q\propto a^{2}/{\left(w-w_{SBS}\right)}^{2}$, as shown in Figure 2c, where $Ba^{2}/{\left(w-w_{SBS}\right)}^{2}$ is used to fit the *Q*-value curve with the fitting constant B. The *Q* factor here are ultra-high that more than 10^10^. The *Q* value is calculated using the following formula:

(1) $$Q=\frac{F_{f}}{FWHM},$$

where $F_{f}$ is the frequency of the peak in transmission and *FWHM* (full width of half maximum) is the frequency width at half value of transmission peak.

**Figure 3:**
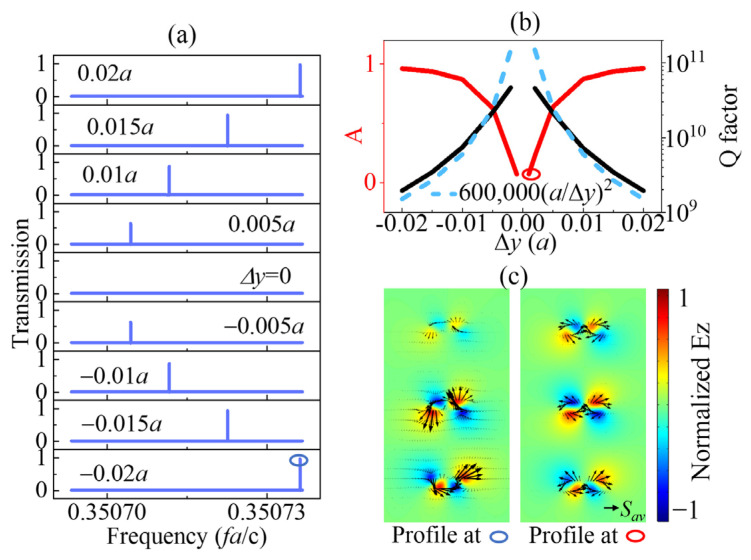
Evolution of (**a**) transmission spectral profile and (**b**) *Q* factors (black line) and amplitude (*A*, red line) of the SP SBS mode with different Δy values for h = 0.2a, w = 0.515525a. (**c**) Electrical field patterns in *z*-direction of the defect cavity in the SBS point and QSBS point.

Next, the evolution of transmission, field profiles, and *Q* factors of the counterpart named SP SBS mode is given in Figure 3. In the previous section, the geometric parameter w was tuned to produce AC SBS, and no structural symmetry was broken until the SP SBS mode was discussed with the parameter Δy induced. The transmissions in Figure 3a indicate that the amplitudes of the peaks decreased to 0 with the absolute value of Δy closing to 0. As is known, the infinite *Q* factor is one of the most specific characteristics of the BIC. Then, the corresponding tendencies of the *Q* factors and amplitude are detailly shown in Figure 3b. The amplitudes decreased to a small value, while the *Q* factors increased dramatically with Δy closing to 0. For the *Q* factor, it increased quadratically concerning (a/Δy) ($Q\propto {\left(a/\Delta y\right)}^{2}$), as shown in Figure 3b, where $B{\left(a/\Delta y\right)}^{2}$ was used to fit the *Q*-value curve with the fitting constant B. The *Q* factor here was also as high as more than 10^10^. To compare the QBIC and BIC points, the field patterns and Poynting vectors at the blue circle point with Δy = −0.02a and the red circle point with Δy = 0.001a are illustrated in Figure 3a and Figure 3b, respectively. These patterns and vectors differed from those shown in Figure 2b. The field pattern and Poynting vector in the left side panel of Figure 3c were much weaker in the upper rectangular column than the other two. The topological symmetry was broken to provide a radiation tunnel for the peak is near 1 with Δy = −0.02a. However, the topological symmetry was reserved to constrain the energy in the cavity in the right panel of Figure 3c, in which the corresponding amplitude was 0.06 with Δy = 0.001a.

## 3. Results

Sensitivity *S* is a very important parameter in sensor research, which reflects the change of the sensor with detection parameters. The general definition of sensitivity is *S* = ∆*λ*/∆*n*, where ∆*λ* refers to the change in wavelength under different refractive indices, ∆*n* is the change in refractive index. Another very important parameter in the sensors is the quality factor *FOM*, which is generally defined as:

(2) $$FOM=S\times \frac{Q}{\lambda_{res}}=\frac{S}{FWHM},$$

where *S* is the sensitivity of the sensor mentioned earlier, *Q* is the quality factor of the resonator, $\lambda_{res}$ refers to the resonant frequency of the resonator, and *FWHM* is the frequency width at half value of transmission peak.

**Figure 4:**
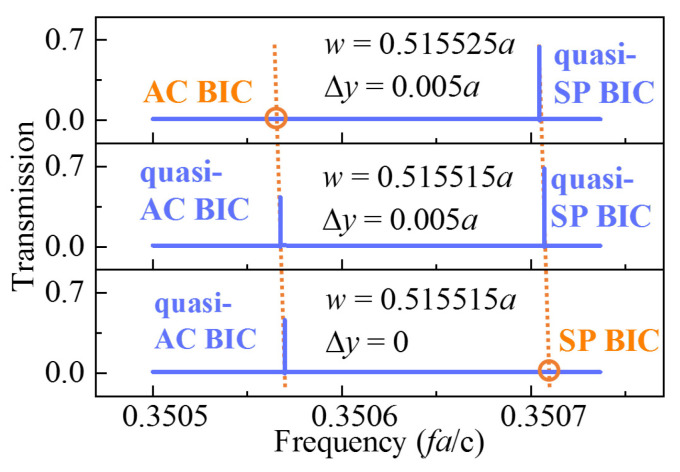
Evolution of transmission spectral profile of the two SBS modes with different w and Δy values when h = 0.2a.

From the tuned geometric parameters in the previous sections, the related two parameters w and Δy can control the AC BIC and SP SBS independently. By adjusting the parameters, we can obtain three kinds of transmission lines in Figure 4. The first one, as in the top panel, shows the result that there was only SP QSBS in the transmission spectra with w = 0.515525a, Δy = 0.005a. The second one is shown in the middle with both SP QSBS and AC QSBS in the transmission when w = 0.515515a, Δy = 0.005a. The last one in the bottom panel had only AC QSBS in the transmission spectrum, in which w = 0.515515a, Δy = 0. The top and the bottom transmission line can be used as ultra-wide FSR high *FOM* sensors, while the middle panel case can function as multi-mode sensing. For the possibility of fabricating the sensor, we considered that the sensor was applied in the frequency region of THz, and the lattice of the photonic crystal was confirmed as a = 1 mm. Then, the other geometric parameters of h = 0.2 mm and w were established.

**Figure 5:**
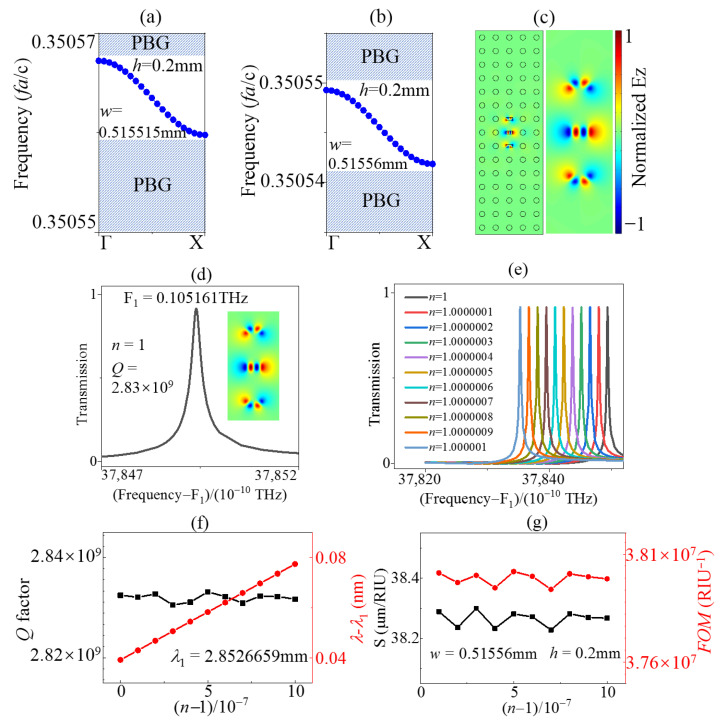
TE band maps of the AC BIC mode for h = 0.2 mm, (**a**) w = 0.515515 mm and (**b**) w = 0.51556 mm in the bandgap along the Γ-X direction. (**c**) Electrical field pattern in z-direction of the AC SBS mode when h = 0.2 mm, w = 0.515515 mm. (**d**) Transmission line of the AC SBS mode when h = 0.2 mm, w = 0.51556 mm. Evolution of (**e**) transmission spectral profile, (**f**) *Q* factors, peak frequencies, and (**g**) *S*, and *FOM* of the AC SBS mode with different n values when h = 0.2 mm, w = 0.51556 mm.

The band maps of the even AC SBS mode with different w values are shown in Figure 5a,b. It is evident that this mode resided within the band gap, effectively avoiding interference from background light. The field pattern in Figure 5c is consistent with the even mode pattern in Figure 1d of AC BIC. The transmission in Figure 5d shows a very sharp Lorentz shape line in which the *Q* factor reached high to 2.83 × 10^9^ with h = 0.2 mm, w = 0.51556 mm. For the lone AC BIC mode in the band gap region, no background interference resulted in a relatively broad band suitable for various sensor applications. In Figure 5e, the peak frequencies decrease with the permittivity of the background tuned manually. Meanwhile, the evolution of *Q* factors and peak frequencies in Figure 5f demonstrate that the *Q* factors had minor fluctuation and the frequency can be varied linearly with the permittivity. The evaluation parameters *FOM* and *S* were obtained by the equation of (2) in Figure 5g. The values of *FOM* 10^7^ and *S* 38.25 µm/RIU were very high compared to the current research results.

**Figure 6:**
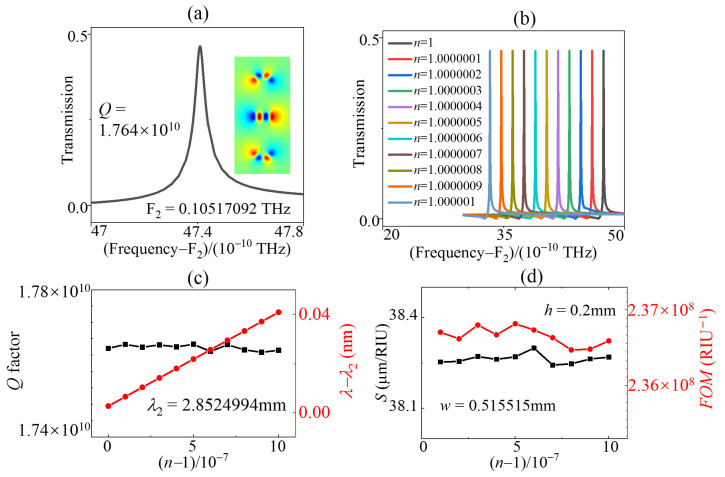
(**a**) Transmission line of the AC SBS mode when h = 0.2 mm, w = 0.515515 mm. Evolution of (**b**) transmission spectral profile, (**c**) *Q* factors, peak frequencies, (**d**) *S*, and *FOM* of the AC SBS mode with different n values when h = 0.2 mm, w = 0.515515 mm.

To further investigate the impact of varying the parameter w on the properties of AC SBS sensors, we conducted calculations and analyzed the sensor characteristics at w = 0.515515a. Comparing the amplitudes of transmission in Figure 6b to those in Figure 5d, we observed a significant reduction (0.464), albeit with an accompanying increase in the *Q* value by an order of magnitude. The evolution of *Q* factors and peak frequencies with different n values in Figure 6c indicates that the *Q* factors exhibited minor fluctuations, and the wavelength can be linearly varied. These observations are consistent with those depicted in Figure 5f. The evaluation parameters, *FOM* and S in Figure 6d, demonstrate that the values of *FOM* 10^8^ were one order of magnitude higher than those of w = 0.51556a (Figure 5g). However, there was no significant change in sensitivity S. This indicates that, for the same structural mode, altering the parameters did not lead to a considerable change in sensitivity *S*. On the other hand, *FOM* was highly influenced by the *Q* value, and modifying the *Q* value can effectively alter *FOM*.

**Figure 7:**
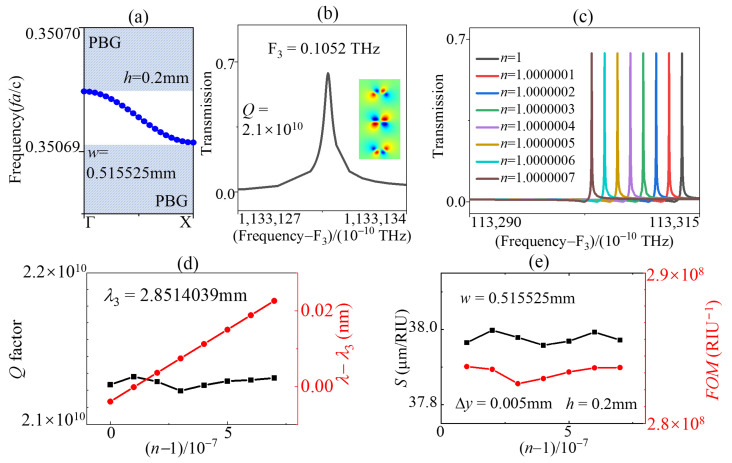
(**a**) TE band maps of the defect cavity modes for h = 0.2 mm, w = 0.515525 mm in the bandgap along the Γ-X direction. (**b**) Transmission line and electrical field pattern in *z*-direction of the SP SBS mode for h = 0.2 mm, w = 0.515525 mm, and Δy = 0.005 mm. Evolution of (**c**) transmission spectral profile, (**d**) *Q* factors, peak frequencies, (**e**) *S*, and *FOM* of the SP SBS mode with different values of n for h = 0.2 mm, w = 0.515525 mm, and Δy = 0.005 mm.

The other single SBS condition in Figure 4 is the SP SBS, which was protected by topological symmetry. When the geometric parameters h = 0.2 mm and w = 0.515525 mm were fixed, the transmitting tunnel was opened by breaking the symmetry with offset parameter Δy = 0.005 mm. From Figure 2, we know the peak of transmission disappeared at the AC SBS point ($w_{\mathrm{SBS}}$ = 0.515525a). Only a single SP QSBS peak appeared in the transmission spectra due to symmetry broken. The band gaps and the field pattern in Figure 7a,b illustrate that the operating mode was odd in the band gaps, as shown in Figure 1d. The *Q* factor of the SP QSBS in Figure 7b was 2.1 × 10^10^ and the amplitude was 0.64 and both are higher than that of AC QSBS in Figure 6a. The transmissions, the evolution of *Q* factor, *S*, and *FOM* varying with refractive were introduced from Figure 7c–e. The difference from the AC QBIC sensor in Figure 6 was larger values of the peaks of transmission, the operating frequency, *Q* factors, and *FOM*, and a smaller value of *S* = 38. This shows that by adjusting the parameters w and Δy, we can change the working mode, frequency, *FOM*, signal amplitude, etc., of the single-mode sensor.

**Figure 8:**
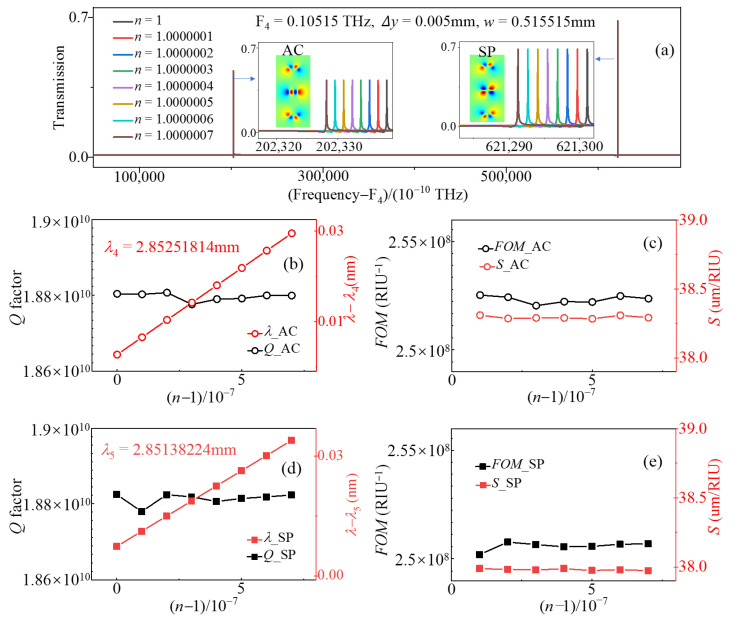
(**a**) Evolution of transmission spectral profile with different n values when h = 0.2 mm, w = 0.515525 mm, Δy = 0.005 mm. Evolution of (**b**) *Q* factors, peak frequencies, (**c**) S, and *FOM* of the AC SBS mode and (**d**) *Q* factors, peak frequencies, (**e**) *S*, and *FOM* of the SP SBS mode with different n values when h = 0.2 mm, w = 0.515525 mm, Δy = 0.005 mm.

In the more common scenario, when both parameters Δy ≠ 0 and w ≠ 0.515525 mm, the transmission exhibited two peaks simultaneously at different frequencies. When h = 0.2 mm, w = 0.515515 mm, Δy = 0.005 mm, both AC QSBS and SP QSBS were in the transmission spectra. The transmission spectra, the evolution of *Q* factors, *S*, and *FOM* varying with the refractive index of a dual-bands sensor are shown in Figure 8. The *Q* factors of the two modes were larger than 10^10^, inducing an ultra-large *FOM* that more than 10^8^. The sensitivity of the AC SBS mode and the SP SBS mode was 38 and 38.3 µm/RIU in the terahertz wave band, respectively. It can be utilized for ultra-sensitive multi-parameter measurements. In addition, the dual-band sensor can also be transformed back into a single-band sensor by adjusting the parameters w and Δy.

Compared to traditional 2D PhC activity sensor structures, most of them have larger sizes, typically exceeding 15 × 15 dimensions [50,51]. The *Q* factor generally does not exceed 10^7^, and the sensitivity remains below 10,000 nm/RIU [50,51]. However, in this study, the 2D PhC cavity structure demonstrated remarkable performance with an ultra-compact size of 5 × 15, achieving a Q value of more than 10^10^ and a sensitivity of 383,000 nm/RIU. Furthermore, it exhibited a high signal-to-noise ratio and an exceptionally wide FSR. These achievements open up possibilities for compact, high-sensitivity biosensing, multi-wavelength sensing, and other promising applications.

## 4. Analysis of the Effect of Fabrication Error and Experimental Scheme

The accuracy of mechanical processing will inevitably affect the sensor parameter indicators. Therefore, it is important to analyze the impact of fabrication errors and determine executable solutions before conducting experiments. In general, the machining accuracy can reach 0.002 to 0.001 mm [52]. We studied the changes in sensor parameters under an error of 0.002 mm. For the SP SBS mode, the accuracy error of Δy will affect the *Q* factor, as $Q\propto {\left(a/\Delta y\right)}^{2}$. Let $Q=B{\left(a/\Delta y\right)}^{2}$, then

(3) $$\delta Q=B\frac{d{\left(a/\Delta y\right)}^{2}}{d\Delta y}\delta \Delta y=-2Q\frac{\delta \Delta y}{\Delta y}.$$

In Figure 3b, when Δy = 0.005a and 0.009a, *Q* = 2.1 × 10^10^ and 9 × 10^9^, respectively, where a = 1 mm. Therefore, when Δy = 0.007 mm, even with an error of δΔy = 0.002 mm, the variation of *Q* will be

(4) $$\frac{\delta Q}{Q}=-2\frac{\delta \Delta y}{\Delta y}=-57\%,$$

thus, the value of *Q* has an evident fall, but it is still high in between 0.903 × 10^10^ and 3.87 × 10^9^, which means the sensor can still has high sensitivity and *FOM*.

**Figure 9:**
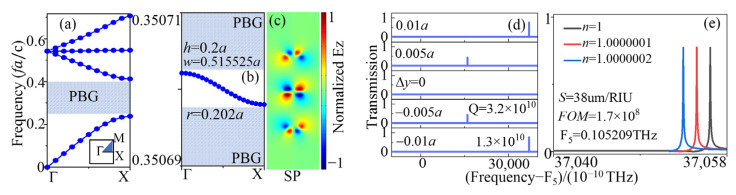
(**a**) TE band maps of the perfect PhC with r = 0.202 mm, a = 1 mm. (**b**) TE Band map and (**c**) electrical field pattern in z-direction of the SP SBS mode with r = 0.202 mm, h = 0.2 mm, w = 0.515525 mm. (**d**) For r = 0.202 mm, h = 0.2 mm, w = 0.515525 mm, the evolution of transmission spectra of the SP SBS mode with different Δy values. (**e**) For r = 0.202 mm, h = 0.2 mm, w = 0.515525 mm, and Δy = 0.01 mm, the evolution of transmission spectra of the SP SBS mode with different n values.

The effect of the radius r of the silicon column with a fabrication error of 0.002 mm on the parameters of the SP SBS mode sensor was studied. Comparing Figure 9a with Figure 1b, when r increased from 0.2 mm to 0.202 mm, the band gap range of the perfect photonic crystal became 0.2379–0.41229, with a slight red shift in frequency and no significant change in band gap width. With the same h and w values in Figure 7a and Figure 9b, the increase in r induced a slight blue shift in the frequency of the band map of the SP SBS mode. Comparing Figure 9c with Figure 1d and Figure 9d with Figure 3a, the change of r had no evident effect on both the mode field distribution and the evolution of the transmission spectrum. Figure 9e shows the parameters of the SP SBS mode sensor with changed r, the *FOM*, and the sensitivity *S* showed only slight changes compared to that in Figure 7. According to the above, we can see that the SP SBS mode sensor had strong robustness.

**Figure 10:**
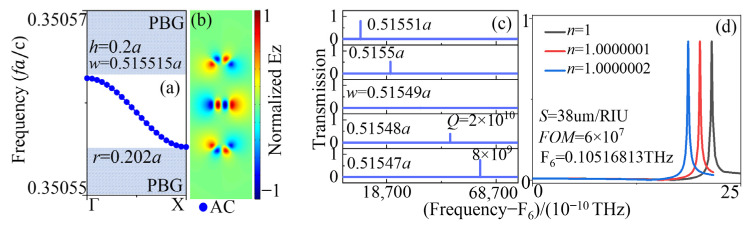
(**a**) TE band map and (**b**) electrical field pattern in z-direction of the AC SBS mode with r = 0.202 mm, h = 0.2 mm, w = 0.515515 mm. (**c**) For r = 0.202 mm, h = 0.2 mm, the evolution of transmission spectra of the AC SBS mode with different w values. (**d**) For r = 0.202 mm, h = 0.2 mm, w = 0.515515 mm, the evolution of transmission spectra of the AC SBS mode with different n values.

Moreover, we investigated the effect of the radius r of the silicon column with an error of 0.002 mm. As shown in Figure 10, the situation was the same with the SP SBS mode sensor except for two things. The first one was that the increase in r induced a slight red shift in the frequency of the AC SBS mode in Figure 10a, not as the blue shift in Figure 9. The second one was that the value of $w_{SBS}$ changed from 0.515525a in Figure 2a to 0.51549a in Figure 10c, which caused a variation in *Q* value, resulting in the *FOM* dropping by an order of magnitude. Compared with the SP SBS mode, the AC SBS mode sensor was more affected by changes in r, and if the w value can be adjusted to be closer to $w_{SBS}$, it can compensate for the loss of the *Q* value.

**Figure 11:**
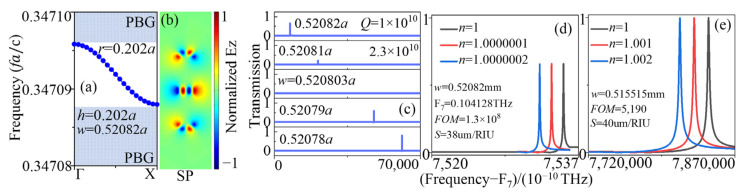
(**a**) TE band map and (**b**) electrical field pattern in z-direction of the AC SBS mode with r = 0.202 mm, h = 0.202 mm, and w = 0.52082 mm. (**c**) For r = 0.202 mm, h = 0.202 mm, the evolution of transmission spectra of the AC SBS mode with different w values. For r = 0.202 mm, h = 0.202 mm, (**d**) w = 0.52082 mm and (**e**) w = 0.515515 mm, the evolution of transmission spectra of the AC SBS mode with different n values.

Additionally, we studied the effect of the parameters h and w with an error of 0.002 mm on the AC SBS mode sensor. In Figure 2c, when w = 0.51551a and 0.5154a, *Q* = 1.3 × 10^10^ and 1.9 × 10^8^, respectively, where a = 1 mm. The error in w value of 0.00011 mm resulted in a change of two orders of magnitude in *Q* value, let alone an error of 0.002 mm. The fitted B of the AC SBS mode is 3 in Figure 2c, which is much smaller than that of the SP SBS mode in Figure 3b. So, the AC SBS mode was very sensitive to geometric parameter w. In Figure 11, the value of h increased to 0.202 mm, and the value of $w_{SBS}$ increased to 0.520803a. The value of w is close to $w_{SBS}$ in Figure 11d, the *FOM* and *S* are as high as that in Figure 6. However, the w value in Figure 11e is the same as that in Figure 6, with a 0.002 mm error in h, resulting in the *FOM* value as low as 5019, reducing it by five orders in magnitude. Therefore, the ability to adjust the w value is very important for the practical application of the AC SBS mode sensor. 

**Figure 12:**
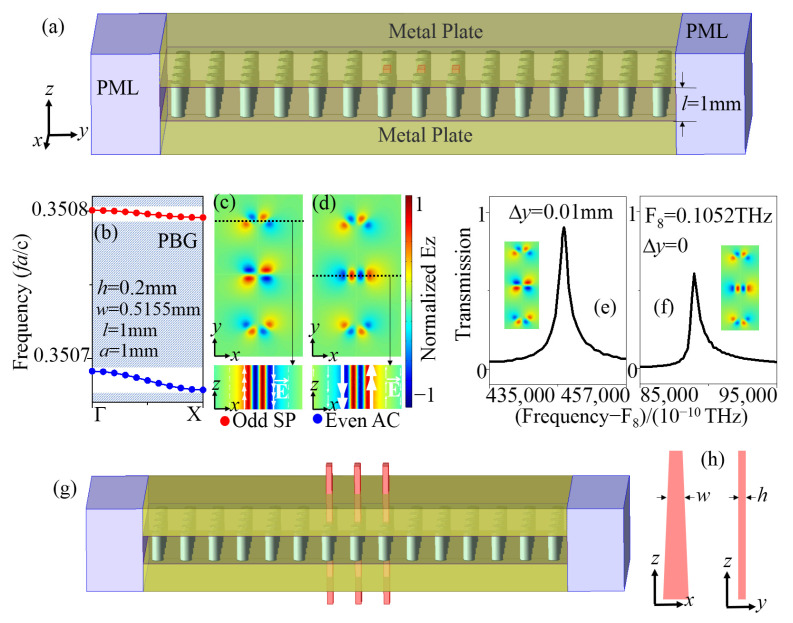
(**a**) Schematic of the 3D PhC cavity structure. (**b**) TE band maps of the SBS modes in 3D PhC cavity along the Γ-X direction with h = 0.2 mm, w = 0.5155 mm, l = 1 mm, and Δy = 0. Electrical field patterns in z-direction of the (**c**) odd SP SBS mode and (**d**) even AC SBS mode with h = 0.2 mm, w = 0.5155 mm, l = 1 mm, and Δy = 0. For h = 0.2 mm, w = 0.5155 mm, and l = 1 mm, the transmission spectra of (**e**) SP QSBS with Δy = 0.01 mm, and (**f**) AC QSBS with Δy = 0. (**g**) Schematic of the 3D PhC cavity structure with 3 trapezoidal columns. (**h**) Schematic of the trapezoidal column.

To construct w adjustable structure, we proposed using long trapezoidal columns instead of rectangular columns in a three-dimensional model with extremely short column lengths in the *z*-axis direction to achieve adjustment of the w value. We first discussed the construction of 3D models. In this study, the input electromagnetic wave was a TE-polarized wave, with the electric field vector oriented perpendicular to the direction of propagation (in the y-direction). It can be directly input at the wave port. To approach the 2D structure more precisely, very long dielectric columns are usually required, which can be challenging to achieve. Instead, a proposed scheme involves the introduction of two metal plates, creating mirrors for the dielectric columns and electromagnetic waves, thereby achieving an equivalent 2D PhC. To mitigate the loss caused by the metal, silver can be used as the material due to its favorable properties. To prevent oxidation of the silver, an organic film can be deposited on the silver plates. This approach significantly reduces the required length of the dielectric columns in z-direction. The three-dimensional model, depicted in Figure 12a, illustrates this concept with a length (l) of 1 mm. The photonic crystal structure and other parameters in the figure remain the same as that in Figure 1a. A TE electromagnetic wave was excited or input from the input port (left port), guided by the metal plates and the PhC, form resonance in the PhC cavity, and finally went to the output port (right port). Within the resonant cavity, the distribution of the electromagnetic field can be approximated as a TE wave. The band map in Figure 12b displays the odd SP SBS mode (red line) and the even AC SBS mode (blue line) in the 3D cavity. It is similar to the band map of the 2D structure in Figure 1c, except for a minor blue shift in frequency. The frequency shift was understandable because the metal material introduced leads to a change of the effective refractive index of the structure. The electric field patterns in the x-y plane of the two SBS modes in the 3D cavity, shown in Figure 12c,d, are the same as those in the 2D cavity in Figure 1d. The electric field patterns in the x-z plane of the two SBS modes in the 3D cavity, also depicted in Figure 12c,d, illustrate the distribution of the electric field in the z-direction. The transmission spectra of the two SBS modes in the 3D cavity are presented in Figure 12f,h. This demonstrates that it is feasible to reduce the length of the dielectric column by utilizing the metal-plate-assisted construction.

Finally, in order to adjust the w value, the rectangular column was designed as a trapezoidal column whose x-z plane was trapezoidal, as shown in Figure 12h. The upper short side of the trapezoid had a length of 0.505 mm, and the lower long side had a length of 0.525 mm, which means the adjustment range of the w value was 0.505–0.525 mm, while h remained fixed. The length of the trapezoidal column in the z-direction was 100 mm, and the part of the trapezoidal column in the resonant cavity could be approximated as a rectangular column. By adjusting the trapezoidal column up and down, the w value can be adjusted to solve the problem caused by insufficient machining accuracy. With this scheme, machining accuracy to 1% is feasible.

## 5. Conclusions

In conclusion, this study introduced a top-bottom symmetric cavity consisting of three rectangular columns with high permittivity. It exhibited both an SP SBS mode and an AC SBS mode located within the band gap, with a period size of 5 × 15. By adjusting the parameters w and Δy, the AC SBS and SP SBS can be converted into QSBS, respectively. This means that the two SBS modes within the band gap can be individually modulated, resulting in a single AC QSBS or SP QSBS in the transmission spectrum. By tuning w and Δy, it was also possible to obtain both QSBS modes simultaneously in the transmission spectrum. Hence, this cavity can function as a single-band sensor or a dual-band sensor by manipulating the aforementioned parameters. For the single-band sensor configuration, the frequency and figure of merit (FOM) can also be adjusted. The achieved *Q* factors of the QSBS modes can exceed 10^10^. The obtained bulk sensitivity was 38 µm/RIU with a high signal-to-noise ratio in the terahertz wave band, and a record-breaking figure of merit of 2.8 × 10^8^ RIU^−1^ was achieved. The effect of fabrication error on the performance of the proposed structure was also discussed, showing feasibility for applications. For experimental realization, a 3D schematic was presented. These accomplishments open up avenues for compact, high-sensitivity biosensing, multi-wavelength sensing, and other promising applications.

## Data Availability

The research data will be supplied with reasonable request.
